# Islet size, cellular composition, and spatial location are interconnected in the human pancreas

**DOI:** 10.64898/2026.10.01.756003

**Published:** 2026-11-04

**Authors:** Sean D. Dufault-Hunter, Vipul Periwal, Manami Hara

## Abstract

Stem cell-derived islets (SC-islets), a promising therapy for type-1 diabetes, are often dissociated and reaggregated into standardized sizes. However, the relationship between endocrine cell composition and size has not been precisely defined in native human islets. In this study, we examined relationships between islet size, composition, and spatial distribution throughout the whole pancreata of 10 well-characterized and biometrically diverse donors. Insulin, glucagon, and somatostatin stains were used to identify the major endocrine cell types. Islets ranging in size from non-islet single cells and small clusters (niSSCs) to >500 μm were analyzed. Islet composition strongly correlated with islet size, with large islets having increased alpha-cell fractions. Delta-cell fraction was consistent across sizes. Approximately 80% of the total endocrine area was contained in islets positive for all three hormones. Spatial analysis additionally revealed clustering among niSSCs, tri-hormone-positive islets, and cells/clusters composed of a single cell type.

## Full Text Availability

The license terms selected by the author(s) for this preprint version do not permit archiving in PMC. The full text is available from the preprint server.

