# Supplementary material for: Islet size and spatial organization govern endocrine cell composition throughout the human pancreas
Source: bioRxiv. 2026 Oct 7:2026.10.01.756003. Preprint. [Version 1] doi: 10.64898/2026.10.01.756003 (PMC13691936; doi:10.64898/2026.10.01.756003)
Supplement: Supplementary file 1 [file media-1.pdf]

Contents

Supplementary Table 1: Donor characteristics.

Supplementary Table 2: Empirical per-islet composition.

Supplementary Figure 1: Size distributions of pancreatic islets.

Supplementary Figure 2: Per-subject composition analysis.

Supplementary Figure 3: Representing islets with point patterns.

Supplementary Figure 4: Inter- and intra-donor variance in proportions.

Supplementary Table 1: Donor characteristics.

ID	Age	Sex	BMI	Ethnicity
**1**	38	M	31.4	White
**2**	43	M	30	White
**3**	43	M	27.9	White
**4**	48	M	16.4	White
**5**	39	M	25.1	AA
**6**	43	M	25.3	White
**7**	43	M	34.8	-
**8**	44	M	23.8	Arab
**9**	44	M	36	White
**10**	39	M	24.4	White

Supplementary Table 2: Empirical per-islet composition.

IED (um)	INS Fraction (%)	GCG Fraction (%)	SST Fraction (%)
**(10,21]**	56.84±9.15	25.11±8.66	18.05±3.37
**(21,30]**	60.05±8.19	22.57±7.49	17.39±3.59
**(30,42]**	62.96±7.89	20.44±6.38	16.61±4.60
**(42,60]**	62.76±8.30	21.20±5.97	16.05±6.92
**(60,85]**	59.28±9.25	24.85±6.95	15.87±9.73
**(85,120]**	54.38±8.65	29.54±7.35	16.08±11.66
**(120,170]**	47.90±9.21	35.31±8.40	16.79±13.58
**(170,241]**	42.84±9.75	39.95±9.53	17.21±14.72
**(241,340]**	39.79±10.47	43.03±11.56	17.18±15.37
**(340,480]**	43.96±12.3	38.43±16.01	17.61±12.70

Data are presented as the unweighted mean between the ten pancreata examined ± one standard deviation.

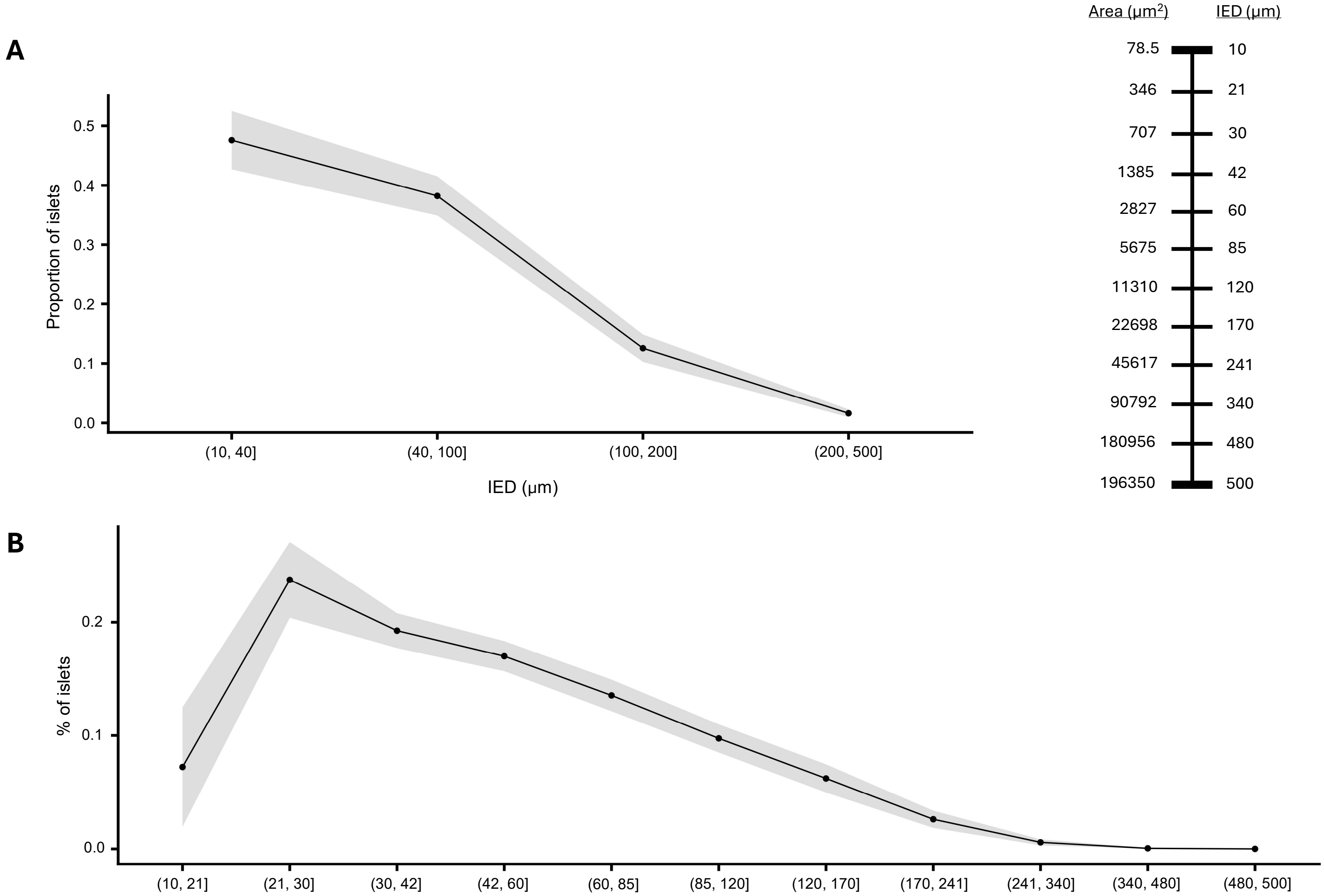

Supplementary Figure 1: Size distributions of pancreatic islets. (**A**) Distribution of islets across wider bins. Points represent the mean value between all subjects; ribbons represent one standard deviation. Area to effective diameter conversion is shown to the side. (**B**) Distribution of islets across narrower bins.

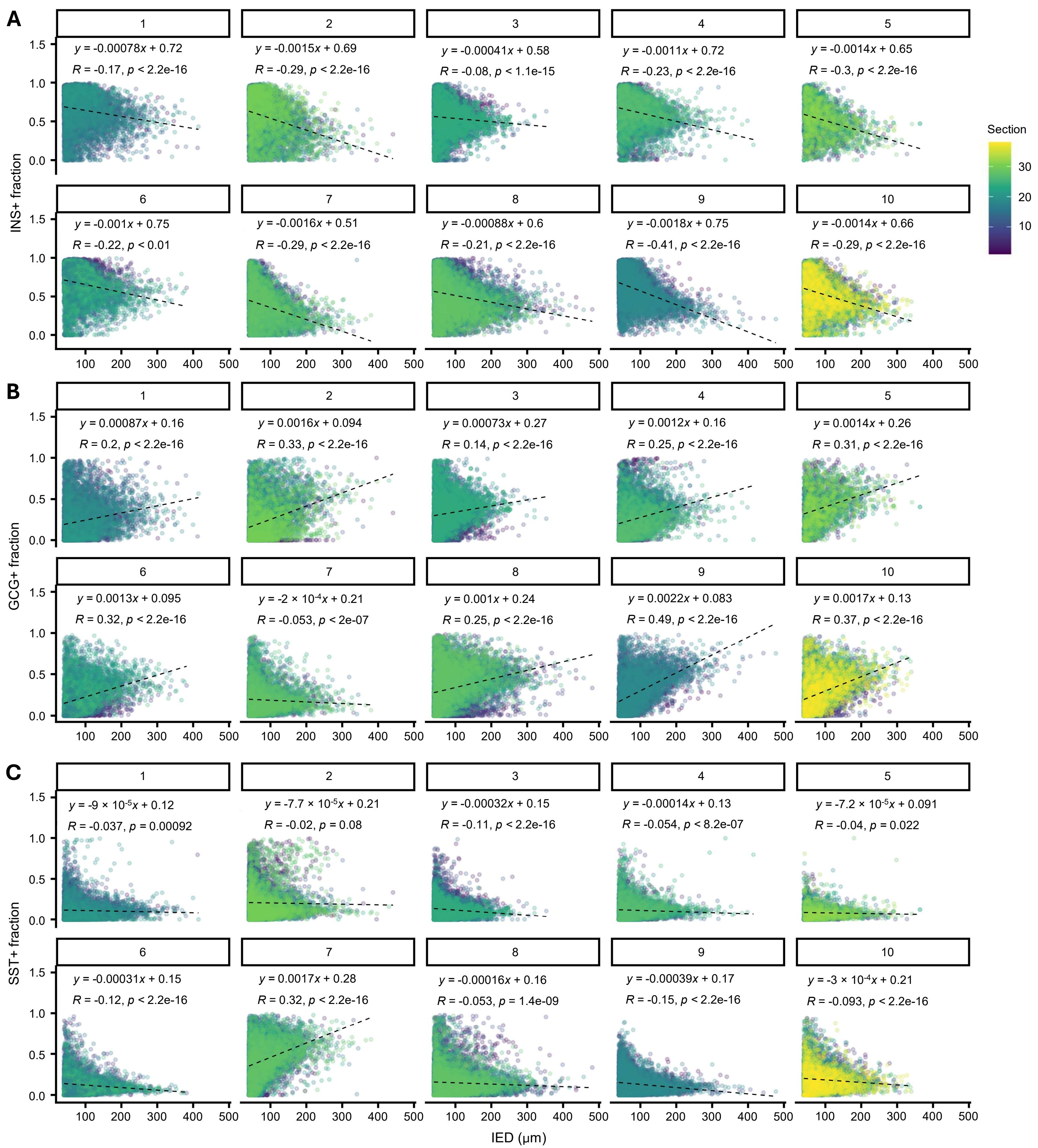

Supplementary Figure 2: Per-subject composition analysis. All islets in each subject are shown. Point color indicates the section the islet is from. The dotted line shows a fitted linear regression, with the formula and correlation coefficient above. IED was used as the predictor for hormone fraction. niSSCs were excluded. (**A**) Insulin fraction. (**B**) Glucagon fraction. (**C**) Somatostatin fraction.

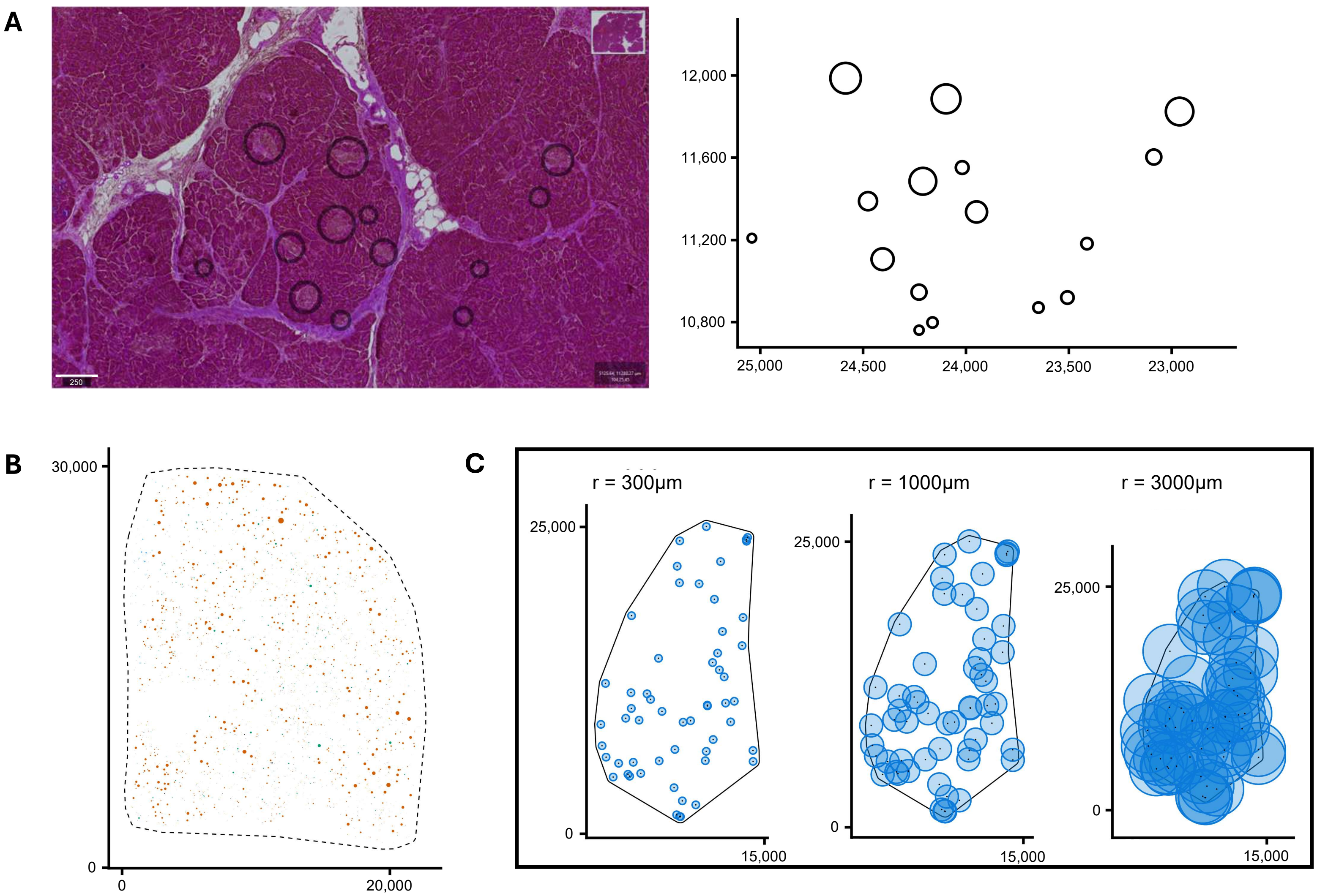

Supplementary Figure 3: Representing islets with point patterns. (**A**) A magnified H&E tissue image and its corresponding point pattern. Circles denote islets and are scaled to IED. (**B**) An example of a point pattern created from the original imaging data. Points are scaled to IED and colored by hormone positivity (see fig. 3). The dotted line represents the computed tissue hull. (**C**) Visualization of Ripley's K/empty-space function. Circles of a given radius are drawn around each point, and the points falling in those circles are counted.

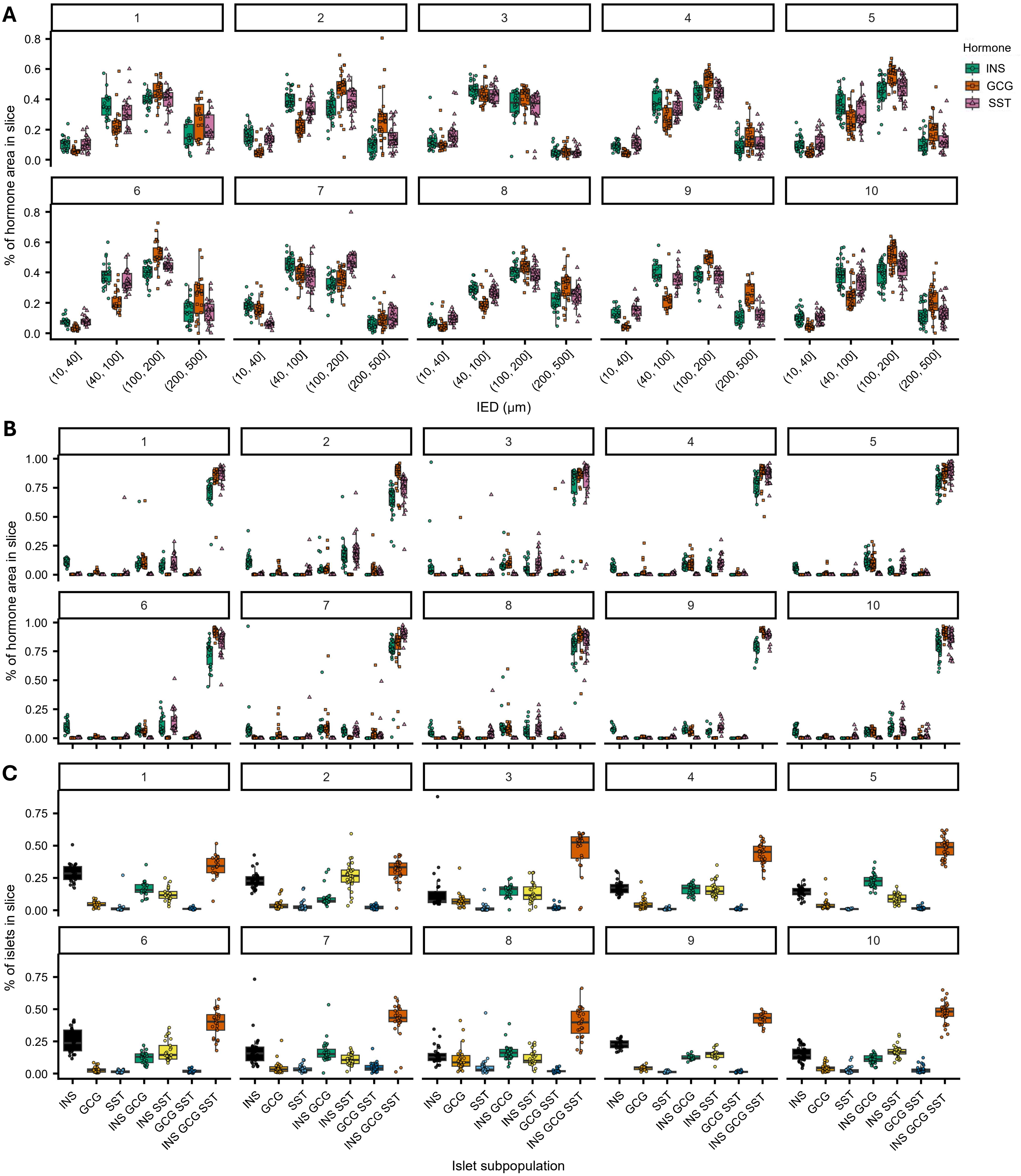

Supplementary Figure 4: Inter- and intra-donor variance in proportions. (**A**) Proportion of total hormone area in IED bins. Points on box-plots represent the mean value calculated from each section. Values are separated by subject (**B**) Proportion of total hormone area in each subpopulation. (**C**) Frequency of each subpopulation.
